# TPO/Mpl Studies in Agnogenic Myeloid Metaplasia

**DOI:** 10.1186/1478-811X-3-4

**Published:** 2005-02-03

**Authors:** Kirugaval C Hemavathy, Kathir Suppiah, Gazala Hashmi, Allan D Novetsky, Jen C Wang

**Affiliations:** 1 Division of Hematology/Oncology, Department of Medicine, Maimonides Medical Center, Brooklyn, New York, USA

## Abstract

**Background:**

Agnogenic myeloid metaplasia (AMM) is one of the Philadelphia chromosome negative myeloproliferative disorder and is diagnosed by hyperplasia of atypical megakaryocytes, hepatosplenomegaly, extramedullary hematopoiesis and bone marrow fibrosis. Fibrosis is considered to be a secondary consequence of enhanced levels of fibrogenic growth factors such as TGF β1, bFGF and PDGF produced by enhanced numbers of megakaryocytes, while the primary cause is considered to be the enhanced proliferation of a defective stem cell. We have previously reported that thrombopoietin (TPO) is elevated in patients with AMM. Others have reported that Mpl protein is decreased in these patients. Since TPO is essential for the development of megakaryocytes, and Mpl protein is the receptor for TPO, we extended the study of TPO/Mpl to in vitro and in vivo cell culture systems to better understand the mechanism that leads to reduced Mpl protein in AMM patients.

**Results:**

Plasma TPO levels were significantly elevated and Mpl protein levels were significantly reduced in AMM patients in concordance with previous studies. Platelet Mpl transcripts in AMM were however similar to those in controls. We also cloned Mpl cDNA from AMM patients and tested for their ability to make functional proteins in vitro and in the in vivo system of 293 T human embryonic kidney cells. Their expression including the glycosylated forms was similar to those from the controls. We also measured the level of translation initiation factor, eIF4E and found it to be increased in patients with AMM demonstrating that the reduced Mpl protein may not be due to translation defects.

**Conclusions:**

Our studies using the in vitro and in vivo systems further confirm that reduced Mpl protein levels are not due to defects in its transcription/translation. Reduced Mpl protein could be due to its increased internalisation owing to enhanced plasma TPO or in vivo intrinsic defects in patients with AMM.

## Background

Agnogenic myeloid metaplasia (AMM), polycythemia vera (PV), essential thrombocythemia (ET) and chronic myelogenous leukemia (CML) are characterized as myeloproliferative disorders (MPDs) arising due to exponential amplification of a haematopoietic stem cell. A typical feature of these disorders is the presence of high number of circulating progenitor cells and their cytokine independent growth in culture. So far, the molecular basis for the disorder has been recognized only for CML and is attributed to the Bcr/Abl or Philadelphia (Ph) chromosome arising due to a translocation event. The aetiology for the other three disorders referred to as Ph negative MPDs is unknown.

AMM, one of the Ph negative MPD is diagnosed by hyperplasia of atypical megakaryocytes, hepatosplenomegaly, extramedullary haematopoiesis and bone marrow fibrosis. Fibrosis is considered to be a secondary consequence of enhanced levels of fibrogenic growth factors such as TGF β1, bFGF and PDGF produced by enhanced numbers of megakaryocytes (MKs), while the primary cause is considered to be the enhanced proliferation of a defective stem cell. What leads to generation of such a defective cell with enhanced proliferation is unknown. We have earlier demonstrated [1], which has later been confirmed [2] that thrombopoietin (TPO) is one of the growth factors whose level is enhanced in patients with AMM. TPO is a haematopoietic growth factor that is essential for megakaryocytopoiesis and thrombocytopoiesis [3]. TPO binds to its receptor Mpl (Myeloproliferative leukaemia), gets internalised [4], and initiates a STAT5 signalling cascade [5], which results in thrombopoiesis. Studies with *TPO *knockout mice have shown reduced numbers of platelets and myeloid progenitor cells confirming the role of TPO. Enhanced expression of *TPO *in mouse by Retroviral/Adenoviral gene transfers and in mice transgenic for *TPO*, the animals developed extramedullary haematopoiesis and splenomegaly due to MK and granulocytic hyperplasia. The severity of the conditions was related to the expression levels of *TPO*, resulting in myelofibrosis under very high expression level [6-8]. These results of *TPO *over expression are similar to the symptoms of AMM and hence indicate that high levels of TPO found in AMM patients could be responsible for the clinical features of AMM. In the mouse knockouts for *Mpl *receptor, there is about 85% reduction in the number of platelets, MKs and other haematopoietic cell types [9] and *Mpl *over expression leads to myeloproliferation [10]. In vitro, *Mpl *antisense oligonucleotides inhibited megakaryocytopoiesis [11]. These reports augment the role of Mpl in megakaryopoiesis. But in contrast to TPO, Mpl protein level is reduced in AMM patients [12]. This abnormality in the TPO/Mpl pathway may depict the clinical features, but is it the cause for MK hyperplasia and the disease? Studies so far have not been conclusive.

According to Taksin et al. [13] defects in the TPO/Mpl pathway are unlikely to be primarily responsible for AMM. Autonomously growing MKs in AMM did not have enhanced *TPO *transcripts and they were not inhibited by TPO antibody. Also no mutations were detected in the *Mpl *gene in these patients, to account for its reduced protein level. The observation of Li et al. [14] that *Mpl *anti-sense oligonucleotide treatment could inhibit the autonomous growth of MKs from ET/PV/AMM in vitro suggests that expression of Mpl is essential for MK autonomy in these disorders. However Moliterno et al. [12,15] have reported reduced Mpl protein level with glycosylation defects in the platelets of patients with these disorders. Although this could be responsible for the impaired TPO-mediated platelet protein tyrosine phosphorylation seen in PV and AMM patients [16], it does not confer with MK autonomy/hypersensitivity for growth in vitro. This reduced expression of *Mpl *in a disorder characterized by MK hyperplasia whose development and differentiation is dependent on this cytokine is paradoxical and needs further investigation.

Hence to better understand the role of TPO/Mpl in AMM, we have extended the study of this growth factor and its receptor gene in these patients. Our studies reveal enhanced plasma TPO level, reduced endogenous level of Mpl protein in the platelets of patients with AMM and absence of any co-relation between TPO level and Mpl expression. We have cloned *Mpl *cDNA from 15 AMM patients and have found that it has full potential for expression similar to controls both in vitro and in the in vivo heterologous system of 293 T cells. We also assayed for the level of eIF4E, the translation initiation factor that under normal limiting level favours translation of strong mRNAs such as those of house keeping genes and at higher level, enhances the translation of weak mRNAs such as *Mpl*. AMM patients were found to have elevated eIF4E level.

## Results

### TPO ELISA

**Figure 1:**
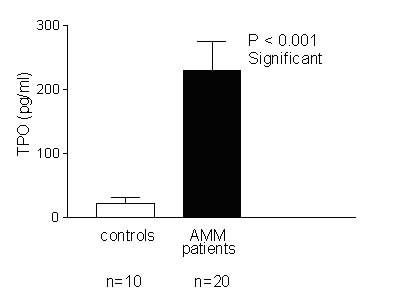
**TPO is elevated in AMM patients. **TPO level in the undiluted Platelet Poor Plasma samples of 10 controls and 20 AMM patients was tested by ELISA. While TPO level was in the range of 0–71 pg/ml in controls (Mean 22.8 pg/ml), it ranged between 0–684 pg/ml in AMM patients (Mean 229 pg/ml). This elevation of TPO level in patients was significant (P < 0.001) compared to controls.

Undiluted platelet poor plasma (PPP) samples from 20 AMM patients and 10 controls were assayed for their TPO levels. Although some patients exhibited low TPO levels, overall, TPO level was enhanced in AMM patients as compared to controls [0–684 pg/ml in AMM patients (with a mean of 229 pg/ml) as compared to 0–71 pg/ml in controls (with a mean of 22.8 pg/ml)]. This increase in TPO was significant (P < 0.001) as determined by t-test (Fig. 1). Minimum detectable dose with the TPO ELISA kit is 2.78–18.5 pg/ml according to the manufacturer. The Optical Density (OD) values for samples were within the range obtained with the highest standard (2000 pg/ml) used in the ELISA. Some samples from controls and patients as well failed to register any OD probably due to very low TPO levels.

### Mpl Expression is reduced in platelets of patients with AMM

**Figure 2:**
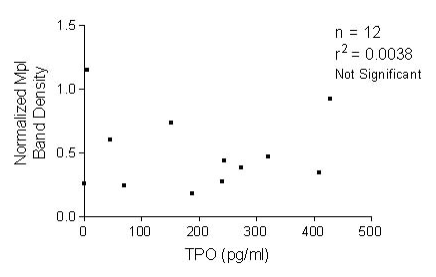
**TPO level is not co-related to platelet Mpl protein level. **Plasma TPO levels and platelet Mpl protein levels from 12 AMM patients were compared for co-relation. Statistical analysis revealed absence of any co-relation between the levels of these two proteins (r^2 ^= 0.0038).

**Figure 3:**
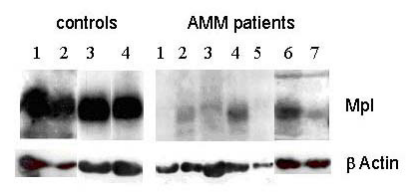
**Mpl protein is reduced in AMM patients. **Platelet lysates from 12 controls and 12 AMM patients in 1X Laemelli buffer were analysed by 6% SDS PAGE. Mpl antibody raised against the full-length protein was used for western blot analysis. The blots were re-probed with β Actin Antibody for normalization. This representative autoradiogram shows the results for 4 controls and 7 AMM patients.

**Figure 4:**
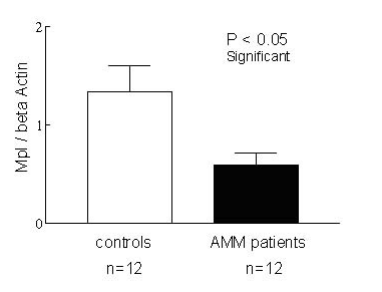
**Densitometric scanning results of Mpl expression in platelets. **The intensity of the bands on the Mpl western blot autoradiograph was quantitated using Image J program. The values represent Mpl expression after normalization with β Actin expression assayed for 12 controls and 12 AMM patients. The reduction in Mpl levels in the platelets of AMM patients was statistically significant (P < 0.05).

Platelets obtained from 12 AMM patients and 12 healthy controls were lysed in 1X Laemelli buffer and analysed on SDS PAGE. Mpl antibody that was raised against the full length Mpl protein was used for western blot analysis. This antibody recognizes both glycosylated and non-glycosylated forms of Mpl. The blots were re-probed with β Actin antibody for normalization. The western blots on chemiluminescence detection showed an overall reduction in the expression of Mpl in the platelets of patients with AMM and there was no co-relation (r^2 ^= 0.0038) between TPO level and Mpl levels (Fig. 2). While there was high expression of glycosylated form of Mpl in the controls, the expression of Mpl in the platelets of patients with AMM varied from none to having reduced amounts of both glycosylated and faster moving non-glycosylated forms. A representative blot with results from 4 controls and 7 patients is shown in Fig. 3. The developed blots were analysed using the Image J program to quantitate the intensity of the bands and the values for Mpl expression after normalization against β Actin expression are represented in Fig. 4. Statistical analysis of the values by t-test revealed the reduced Mpl expression to be significant (P < 0.05).

### Mpl RNA level is similar in controls and AMM patients

**Figure 5:**
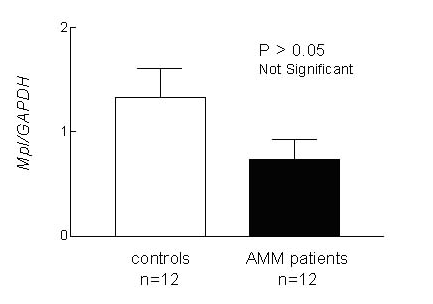
**Transcription of Mpl is similar in AMM and controls. **Total RNA isolated from platelets of 12 AMM patients and 12 controls were subjected to Real-Time RT-PCR using *Mpl *gene specific primers. The values for *Mpl *expression after normalization against *GAPDH *values were found not to be significant (P > 0.05) between the two groups.

Since Mpl protein level was reduced in AMM patients, to know if it was due to reduced transcription of the *Mpl *gene, we compared the levels of *Mpl *RNA in AMM patients and controls by Real-Time RT-PCR. Total RNA from the platelets of 12 controls and 12 AMM patients were used as templates for PCR with gene specific primers. The values were normalized against *GAPDH *expression and are represented in Fig. 5. On statistical analysis (t-test), the difference in *Mpl *expression was found not to be significant (P > 0.05), indicating absence of any transcriptional inhibition of *Mpl *in AMM patients.

### AMM patients have high eIF4E levels

**Figure 6:**
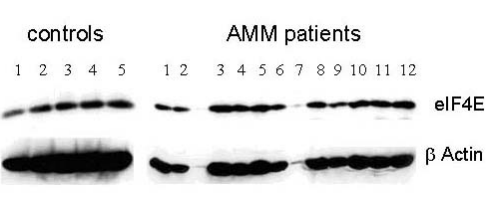
**AMM patients have high levels of eIF4E. **Platelet lysates from 12 controls and 12 AMM patients in 1X Laemelli buffer were analysed by 10% SDS PAGE. Antibody for eIF4E was used at a dilution of 1:1000 for western blot analysis. The blots were re-probed with β Actin Antibody for normalization. A representative autoradiogram including 5 controls and all 12 patient samples is shown in this figure. Platelet sample from patient # 7 has inadequate protein.

**Figure 7:**
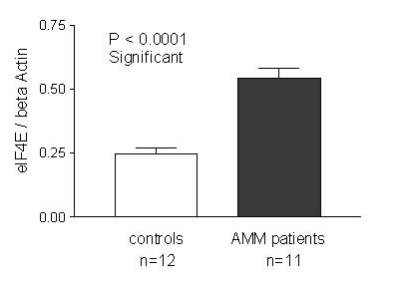
**Densitometric scanning results of eIF4E expression in platelets. **The intensity of the bands on the eIF4E western blot autoradiograph was quantitated using Image J program. The values represent eIF4E expression after normalization with β Actin expression for the 12 controls and 11 patient samples. The increase in eIF4E levels in the platelets of AMM patients was statistically significant (P < 0.0001).

We assayed for the expression of eIF4E in the platelets of 12 AMM patients to see if reduced Mpl protein level could be due to its reduced translation owing to limiting levels of eIF4E, a translation initiation factor that could favour translation of weaker mRNA such as *Mpl *at higher amounts. At limiting levels it favours translation of stronger mRNA over the weaker mRNA. The western blots on chemiluminescence detection showed an overall increase in the expression of eIF4E in the platelets of patients with AMM. A representative blot shown in Fig. 6 includes 5 controls and 12 AMM patients (Platelet sample from Patient # 7 has inadequate protein). The developed blots were analysed using the Image J program to quantitate the intensity of the bands and the values for eIF4E expression after normalization against β Actin expression are represented in Fig. 7. Statistical analysis of the values by t-test revealed the elevated eIF4E expression to be significant (P < 0.0001) and absence of any significant co-relation between the expression levels of Mpl and eIF4E (data not shown).

### The coding region of *Mpl *from AMM patients has no mutations

*Mpl *cDNA was amplified from the platelets of 15 AMM patients along with those from 15 normal controls by RT-PCR, cloned into the Blue Script vector and the recombinant clones sequenced completely. Base changes were detected in some patients and controls as well in the exons 2,3, 4, 6 and 12 but not at the same nucleotide (data not shown). The same changes were however not observed on re-cloning the cDNA of the samples with mutations and sequencing the corresponding genomic region. These base changes were therefore considered to be due to PCR errors and not genuine mutations. Hence *Mpl *gene abnormalities were not detected in AMM patients.

### *Mpl *from AMM patients is able to transcribe and translate in vitro

About 1 μg of the *Mpl *cDNA of 15 controls and 15 AMM patients in the pcDNA3 vector were subjected to in vitro transcription/translation employing the rabbit reticulolysates to see if they were capable of translation in vitro. *Mpl *from AMM patients were able to translate as efficiently as the cDNA from controls revealing their full potential for translation. Also, in the presence of canine microsomal membranes, the translated protein was glycosylated similar to those from the controls.

**Figure 8:**
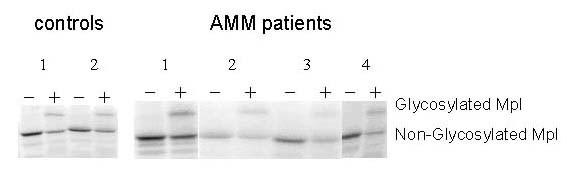
***Mpl *from AMM patients can be translated in vitro similar to controls. ***Mpl *cDNA clones of 15 controls and 15 Patients with AMM in pCDNA3 vector were subjected to in vitro transcription/translation using Promega's TNT Quick Coupled Transcription/Translation Systems with ^35^S Methionine in the absence (-) or presence (+) of canine microsomal membranes. The entire reaction product was loaded onto 6% SDS PAGE and electrophoresed. The dried gel was exposed to X-ray film and a representative autoradiograph with 2 control and 4 patient samples is shown.

**Figure 9:**
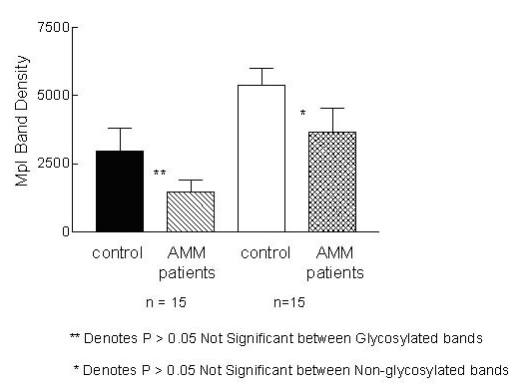
**Densitometric scanning results of *Mpl *in vitro transcription/translation. **The intensity of the bands on the autoradiograph of *Mpl *in vitro transcription/translation was quantitated using Image J program. The values represent Mpl band intensity for 15 samples each of controls and patients. There was no significant (P > 0.05) difference between AMM and controls in the intensity of the non-glycosylated/glycosylated bands.

Results from 2 controls and 4 AMM patients are represented in Fig. 8. The developed autoradiographs were analysed using the Image J program to quantitate the intensity of the bands and the values are represented in Fig. 9. Statistical analysis of the values by t-test revealed absence of any difference (P > 0.05) between cDNA from controls and AMM either in the amount of the translation product or its glycosylation.

### *Mpl *from AMM patients is able to transcribe and translate in vivo

**Figure 10:**
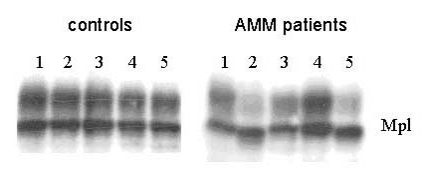
***Mpl *from patients with AMM can be translated in vivo. **Western blot analysis of lysates of 293 T cells transfected with pcDNA3/*Mpl *constructs from 15 AMM patients and 15 controls. Transfection lysates were analysed by 6% SDS PAGE. Blots were probed with Mpl antibody raised against the full length Protein. A representative blot with data for 5 controls and 5 patients is shown in this figure. The second band probably corresponds to non-glycosylated Mpl.

**Figure 11:**
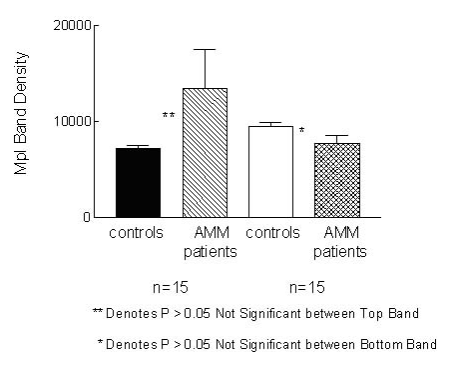
**Densitometric scanning results of western blot of Mpl in vivo expression. **The intensity of the bands for Mpl western blot with in vivo transfection lysates was quantitated using Image J program. No significant (P > 0.05) difference in the intensity of the Mpl bands between AMM and control's cDNA transfection lysates was observed.

*Mpl *cDNA was tested for in vivo expression in the 293 T human embryonic kidney cells. Equal amount of *Mpl *cDNA from 15 AMM/15 controls was transfected into the 293 T cells and Mpl expression analysed by western blot as described for the platelets. The expression level of Mpl appeared to be similar between controls and AMM patients. Fig. 10 is a representative blot depicting results of 5 controls and 5 AMM patients. Two bands were identified. The developed blots were analysed using the Image J program to quantitate the intensity of the bands and the values for Mpl expression are shown in Fig. 11. Statistical analysis of the values by t-test showed no significant difference (P > 0.05) in the level of Mpl expression.

## Discussion

AMM is one of the MPDs for which the molecular defect is unknown. A characteristic feature of the disorder is the presence of atypical megakaryocytes in high numbers and their autonomy or hypersensitivity to growth factors in vitro. Since the haematopoietic growth factor TPO that is essential for the development of megakaryocytes is present at high level in AMM patients, it is of interest to study the role of TPO and its receptor in AMM.

We have earlier reported that AMM patients have elevated plasma TPO level and this is not associated with its enhanced transcription in the bone marrow cells [1,17]. To determine if this enhanced TPO level is due to its receptor abnormalities, we have analysed 12 AMM patients for the presence of Mpl protein in their platelets and found it to be reduced in AMM relative to controls. However, this reduced level was not due to its reduced transcription since Real-Time RT-PCR revealed similar levels of transcripts from AMM patients and controls. To analyse if it could be due to its reduced translation owing to its G-C rich 5' UTR, we estimated the expression of the translation initiation factor eIF4E. The promoter of *Mpl *displays characteristics of weak mRNA that are not translated very efficiently due to G-C rich 5' UTR [18]. The translation initiation factor eIF4E, that is present at low level under normal conditions favours the translation of strong mRNA at these limiting levels. At higher levels, although the translation of strong mRNA is not increased greatly, the translation of weak mRNAs is highly enhanced and this is usually associated with hyper proliferation of cells as in cancers [19]. Hence we assayed for the expression of eIF4E in AMM to see if reduced Mpl protein level could be due to its reduced translation owing to limiting eIF4E level compared to controls. However eIF4E level was rather significantly higher than in controls as seen in other diseases with hyper cellular proliferation. Although this is not direct evidence, at these levels of eIF4E, normal translation of *Mpl *can be expected.

Since there was no significant difference in the *Mpl *RNA level of AMM patients as shown by RT-PCR, we cloned and sequenced *Mpl *gene from these patients to over-rule any structural defects that could prevent translation. Although base changes were observed for some patients and controls as well, on sequencing the corresponding regions of the genomic DNA, the same base changes were not observed. Hence these were not considered to be genuine mutations. Taksin et al. [13] also have reported absence of *Mpl *gene mutations in AMM patients. Hence to date, there are no reports describing Mpl gene mutations in AMM patients although an activating mutation in this gene has been reported by Ding J et al. [20] in a case of familial ET, a rare hereditary MPD. Moliterno et al. [21] have recently reported a single base change polymorphism in the *Mpl *gene of African American patients with MPD that leads to substitution of the 39^th ^amino acid Lysine, with Asparagine in the extra cellular domain of the protein. Patients with this polymorphism exhibited high platelet counts and low platelet Mpl protein. African Americans were however not represented amongst our patients tested. We also performed in vitro transcription/translation with the cloned cDNA from the AMM patients. Similar levels of proteins were formed irrespective of the source of the cDNA, indicating that the cDNA from AMM patients had no structural defects preventing its translation. There was no difference in the extent of glycosylation of the protein either when the reaction was performed in the presence of microsomal membranes capable of post-translational modification in vitro. To further confirm this in vivo, these cDNAs were transfected into 293 T human embryonic kidney cells and the cell lysates analysed by western blots for Mpl expression. Irrespective of the source of the cDNA, similar level of expression of Mpl protein was observed. Two bands were however detected. Since the antibody used can react with both glycosylated and non-glycosylated forms of Mpl, it is likely that the second band corresponds to non-glycosylated form. As the in vivo system is saturated with the over-expressed protein, the system for modification of the protein could be exhausted. Overall, *Mpl *gene from AMM patients does not seem to have any impaired structural features to prevent its transcription/translation.

Our present study shows elevated TPO level; reduced Mpl platelet protein level; absence of *Mpl *transcriptional inhibition and absence of any structural defects in *Mpl *coding region in AMM patients. However, there was no co-relation between TPO level and Mpl expression, similar to the observation of Harrison CN et al. [22] in ET patients.

Plasma TPO level is regulated by platelet counts and MK mass [23]. TPO binds to its receptor Mpl on platelets/MKs and gets internalised. This receptor mediated endocytosis of TPO leads to its catabolism by the lysozymes, without any recycling of the receptors to the surface [24]. In AMM patients there is initial MK hyperplasia and thrombocytosis that leads to enhanced release of growth factors such as Platelet Derived Growth Factor, bFibroblast Growth Factor and Transforming Growth Factor β1 [25-27]. This may lead to increase in *TPO *transcription, since these growth factors have been shown to stimulate the transcription of *TPO *from the bone marrow stromal cells in cultures [28,29]. Our earlier observation that elevated plasma TPO level is not associated with its enhanced transcription in the bone marrow cells [17] could be due to the fact that our cultures predominantly consisted of fibroblasts and not stromal cells.

Enhanced expression of TPO and absence of an accompanying increase in *Mpl *transcription or re-cycling of the internalised receptor may be responsible for persistence of high plasma TPO level in AMM patients. Reduced Mpl level seen in AMM patients may be due to exhaustion of the Mpl pool by binding to the enhanced TPO and its subsequent catabolism. This gains support from an earlier observation in thrombocythemic mice wherein very high and persistent level of TPO due to its induced over expression were found to be associated with reduced platelet Mpl protein without any reduction in *Mpl *transcription [30]. Also in other MPDs such as ET/PV, high plasma TPO level is associated with low Mpl protein level [16,31]. In vivo Mpl post-translational defect may also be responsible for impaired Mpl protein forms and levels in these patients as suggested by Moliterno et al. [15]. Cloning and sequencing of *TPO *5' UTR from these patients may provide additional valuable information on elevated TPO levels since mutations in 5' UTR of *TPO *have been associated with elevated TPO levels without any increase in its transcription in patients with thrombocytosis [32,33].

## Conclusions

Our studies with TPO/Mpl show elevated TPO and reduced Mpl level in AMM patients, without any significant co-relation in their expression levels. *Mpl *cDNAs from AMM patients had full potential for transcription/translation including glycosylation, in vitro and in vivo, similar to those of normal controls. Reduced Mpl protein level may not be due to defects in its transcription/translation but could be due to increased internalisation or intrinsic defects in translation and glycosylation.

## Methods

### Patients

Peripheral blood was obtained from patients with AMM after informed consent and peripheral blood was obtained from normal volunteers to serve as controls. Five volumes of peripheral blood was mixed with 1 volume of ACD buffer pH 4.5–5.5 (2.2% W/V Sodium Citrate; 0.8% Citric Acid; 2.2% Glucose; 50 ng/ml PGE-1) and centrifuged at 250 g for 10 minutes. The cell pellet was diluted 5X with Hank's buffer and subjected to Ficoll/Hypaque density gradient centrifugation. The ring of mononuclear cells was washed 2X with Hank's buffer and processed for isolation of genomic DNA using the Wizard Genomic DNA Purification kit from Promega, Madison, WI, following their instructions. The Platelet Rich Plasma (PRP) supernatant was centrifuged at 800 g for 10' to obtain the platelets. The Platelet Poor Plasma (PPP) was stored at -80°C, and the platelet pellet washed 2X with the Citrate buffer, pH 6.2 (0.05 mmol/L Sodium Citrate; 0.1 mol/L NaCl; 0.14 mol/L Glucose). They were then processed for RNA isolation using the Totally RNA isolation kit from Ambion, Texas, following their instructions or were suspended in 1X PBS and lysed with equal amount of 2X Laemelli buffer (0.125 M Tris HCl pH 6.8; 4% SDS; 0.56 M β Mercaptoethanol; 0.02% Bromophenol Blue; 20% Glycerol) for western blot analysis.

### TPO ELISA

TPO level in the blood plasma was estimated using the Human thrombopoietin Quantikine ELISA kit from R & D Systems, Minneapolis, MN. The assay is based on the two-sided sandwich principle. Standards and undiluted PPP samples from 10 normal controls and 20 AMM patients were incubated in micro-titer Plates whose wells are pre-coated with monoclonal TPO antibody. After washing off the unbound substances, the wells were incubated with polyclonal TPO antibody conjugated with HRP enzyme. After subsequent washes to remove any unbound antibody-enzyme, a substrate solution for HRP was added to the wells. The color developed that is proportional to the amount of TPO from plasma bound to the plate was quantitated using the Opsys MR plate reader.

### Real-Time RT-PCR

Real-Time RT-PCR was performed to determine the expression of *Mpl *in platelets of 12 controls and 12 AMM patients, using the Geneamp 5700 SDS Instrument from Applied Bio-systems, Foster City, CA. Reagent for the assay was the Quantitect RT-PCR kit (Qiagen, Valencia, CA). *Mpl *mRNA in the platelets was reverse transcribed and PCR amplified from 10 ng of total RNA with gene specific primers and a florescent reporter probe that hybridises to the gene in between the two primers. The sequences of the primers were:

MPL FP 5'-AAGTCCTCAGAGAGGACTCCTTTG-3

MPL RP 5'-CAGGCAAGAAGGCTGCAATC-3'

MPL PROBE 5'-FAM CCTCCCAGGCCCAGATGGACTAC-3' BHQ1

The reaction conditions were reverse transcription at 50°C for 30'; 40 cycles of amplification with de-naturation at 94°C for 15", annealing and extension at 60°C for 10".

### Sequence Analysis of *Mpl*

Total RNA extracted from platelets obtained from 15 patients with AMM and 15 normal controls was used to clone the full length *Mpl *cDNA. First strand cDNA was synthesized using the SuperScript First Strand Synthesis System for RT-PCR from Invitrogen, Carlsbad, CA, following their specifications. About 3 μg of total RNA was mixed with 50 ng of random hexamers, and 1 mM dNTPs in 10 μl reaction volume and incubated at 65°C for 5' and left on ice for 1'. A cocktail mix of RT buffer, MgCl_2 _and DTT was added to a final concentration of 1X, 5 mM and 10 mM respectively along with 40 Units of RNase OUT Ribonuclease Inhibitor and incubated at 25°C for 2'. 50 Units of SuperScript II Reverse Transcriptase enzyme was added and incubated at 25°C for 10' and 42°C for 50'. The reaction was terminated by heating at 70°C for 15' and chilled on ice. 2 Units of RNase H was added and incubated at 37°C for 20' to remove the RNA from cDNA: RNA hybrids to increase the sensitivity of PCR from cDNA. *Mpl *was amplified using the Expand High Fidelity PCR kit from Roche, Indianapolis, IN, with gene specific primers containing *Eco*R 1 (FP) and *Xho *1 (RP) Restriction Enzyme sites to enable directional cloning. The Restriction Enzyme sites are indicated in lower case in the Primer sequences given below.

FP 5'-CGgaattcGAAGGGAGGATGGGCTAAGGC-3'

RP 5'-CCGctcgagAGTTTAGCTCTGTCCAGGGAA-3'

The Products were purified using the Wizard PCR Preps DNA Purification System from Promega, Madison, WI, and digested with the Restriction Enzymes, *Eco*R 1 and *Xho *1. The digested products were then cloned into Blue Script vector. The recombinants were checked by restriction analysis and the selected positive clones were sequenced completely at Northwoods DNA Inc., Becida, MN. *Mpl *cDNA were re-cloned from samples that had mutations and sequenced completely. The putative mutations were confirmed by sequencing the corresponding region of the *Mpl *gene from respective samples. For this, the genomic DNA isolated from the peripheral blood samples were PCR amplified with gene specific primers and the PCR products were directly sequenced after purification using the Wizard PCR Preps DNA Purification System from Promega, Madison, WI.

### In vitro transcription/translation of *Mpl*

The *Mpl *cDNA clones in the Blue Script vector were digested with *Eco*R 1 and *Xho *1 and the released *Mpl *cDNA was cloned into pcDNA 3 vector to obtain cDNA with 3' translational signals. These constructs were then subjected to in vitro transcription/translation using the TNT Quick Coupled Transcription/Translation Systems from Promega. Madison, WI, in the presence of ^35^S Methionine from Amersham Biosciences. About 0.5 μg of the constructs were incubated with the TNT Quick-Coupled Master Mix and ^35^S Methionine in the absence or presence of 2 μl of Canine microsomal membranes provided with the TNT Quick Coupled kit, at 30°C for 2 hours. The reactions were terminated by adding 100 μl of 1X SDS loading buffer (50 mM Tris. HCl pH 6.8; 100 mM DTT; 2% SDS; 0.1% Bromophenol Blue; 10% Glycerol) and stored at -20°C. Appropriately translated and processed products were then separated on a 6% polyacrylamide gel. The gel was dried using Model 583 Gel dryer from BIO-RAD, Hercules, CA. The products of translation were visualized by autoradiography of the dried gel. The bands were quantitated using the Image J program.

### Transfections

293 T human embryonic kidney cells were used for the transient transfection studies with pcDNA3/*Mpl *constructs of controls and patients used in the in vitro transcription/translation studies. The cells were seeded onto a 6 well plate at a density of 5 × 10^4 ^cells/well a day before transfection with DMEM medium containing 10% FBS. On the day of the transfection, the cells were washed with DMEM medium without serum and covered with 800 μl of the same. In an eppendorf tube, 1 μg of the DNA was mixed with 100 μl of serum free DMEM medium and 100 μl of DMEM containing 2 μl of Lipofectamine transfection reagent (Invitrogen, Carlsbad, CA) was added to this DNA. The mixture was incubated for 30' at room temperature and then added to the 800 μl of medium covering the cells. After incubating for 5 hours at 37°C the transfection medium was removed and replaced with DMEM medium with 10% FBS. The cells were harvested 48 hours post-transfection. They were washed twice with 1X PBS and the cells dislodged by gentle re-suspension. The cells were collected and centrifuged at 1000 RPM for 10'. The cell pellets were re-suspended in 100 μl of 1 X PBS each and lysed with 100 μl of 2X Laemelli buffer.

### Western Blot Analysis

The Transfection cell lysates/platelet cell lysates in 1X Laemelli buffer were boiled for 5', clarified by centrifugation at 14 000 RPM for 5', loaded onto a 6% SDS PAGE, and transferred onto immobilon nylon membrane. The membrane after transfer was rinsed with 1X PBS and incubated in the blocking solution (5% milk in 1 X PBS; 0.1 % Tween 20) for 1 hr., at room temperature. The membrane was then incubated with 1:10 000 dilutions of Rabbit anti Mpl antibody from Upstate USA Inc. VA, over night at 4°C in the blocking solution. The membrane was then washed 4 X with 1X PBST (1X PBS; 0.1% Tween 20) for 15' each and incubated with 1:5000 dilutions of HRP conjugated anti-rabbit secondary antibodies for 1 hr., at room temperature. Washes with 1X PBST were repeated and the membrane processed for detection using the Western Lightning Chemiluminescence Reagent Kit from Perkin Elmer, Boston, MA, following their recommendations. The bands were quantitated using the Image J program. Similar procedure was followed for the detection of eIF4E protein in the platelets except that a 10% SDS PAGE was used for separation of the proteins. The eIF4E antibody from Transduction Laboratories, CA, was used at 1:1000 dilutions.

## Competing interests

The author(s) declare that they have no competing interests.

## Authors' contributions

KCH performed most of the experiments and was responsible for acquisition, analysis and interpretation of the data in addition to preparation of the manuscript. JCW conceived, designed the study, provided patient samples for the study and critically reviewed the data. KS performed eIF4E western blot. GH performed the early work and helped in preparation of application for grant support. ADN was involved in critical revision of the article, in obtaining patients for the study and obtaining grant support.

All authors read and approved the final manuscript.
